# Valorization of Spent Vetiver Roots for Biochar Generation

**DOI:** 10.3390/molecules29010063

**Published:** 2023-12-21

**Authors:** Sameer Neve, Dibyendu Sarkar, Manas Warke, Teresa Bandosz, Rupali Datta

**Affiliations:** 1 Department of Civil, Environmental, and Ocean Engineering, Stevens Institute of Technology, Hoboken, NJ 07030, USA; sneve@stevens.edu; 2 Department of Biological Sciences, Michigan Technological University, Houghton, MI 49931, USA; mwarke@mtu.edu (M.W.); rupdatta@mtu.edu (R.D.); 3 Department of Chemistry, City College of New York, New York, NY 10031, USA; tbandosz@ccny.cuny.edu

**Keywords:** vetiver, roots, essential oil, pyrolysis, biochar, physicochemical characteristics, circular economy model

## Abstract

Vetiver root is widely used to produce essential oils in the aromatherapy industry. After the extraction of oil, the roots are disposed of as waste. The central objective of this research was to explore the conversion of this waste into a resource using a circular economy framework. To generate biochar, vetiver roots were pyrolyzed at different temperatures (300, 500, and 700 °C) and residence times (30, 60, and 120 min). Analysis showed the root biochar generated at 500 °C and held for 60 min had the highest surface area of 308.15 m^2^/g and a yield of 53.76%, in addition to other favorable characteristics. Comparatively, the surface area and the yield of shoot biochar were significantly lower compared to those of the roots. Repurposing the spent root biomass for environmental and agronomic benefits, our circular economy concept prevents the plant tissue from entering landfills or the waste stream.

## 1. Introduction

Vetiver (*Chrysopogon zizanioides*) is a C4 grass, which has many direct and indirect cultural and industrial applications. Vetiver essential oil extracted from the roots has a woody earthiness that has made it a favorite perfume since ancient times, and it is now a necessary element in almost 20% of all fragrances for men [1]. Commercially available popular fragrances include Chanel’s Sycamore, Miller Harris’ Vetiver Insolent, Acqua Originale, Creed Vetiver Original, and many others. This tropical/subtropical perennial grass grows wild or is farmed in various parts of the world, including Haiti, Indonesia, India, Brazil, China, and La Réunion. There is enormous variability in the output and quality of the essential oil extracted from vetiver roots, ranging from earthy woody balsamic notes to sweet roseate notes, depending on the place of origin, growing practices, climate, and genetic origin [2]. Once the vetiver root is used for oil extraction, the tissue is discarded as waste and sent to landfills. As vetiver shoots are not utilized in the oil extraction process, they also make their way into the waste stream, although some are used for making handicrafts and decorative items.

Among the various strategies being researched and adopted to tackle climate change, a circular economy is one in which the value of goods, materials, and resources is sustained for as long as possible while ensuring minimal waste and resource use. This contrasts with a linear economy, which can be summarized as “take, produce, consume, and dispose of” [3]. Circular economy models have been employed at various levels to prevent environmental damage. Bioenergy and biochar options have been explored widely to manage agricultural and other biological feedstocks to convert waste to a resource [4,5,6]. The circular economy approach is a sustainable solution to the waste management problem, achieved through reducing, reusing, refurbishing, and recycling of waste materials.

Biochar is formed during the pyrolysis of biomass in the absence of oxygen, which results in certain unique physicochemical features, such as high stability due to concentrated aromatic composition, recalcitrance, and high organic carbon content. Biochar is characterized by its high organic carbon content and has a very diverse makeup, with the essential ingredients being carbon, volatile chemicals, mineral content (ash), and moisture. Biochar is primarily amorphous, but it also contains specific crystalline structures depending on the source biomass. Biochar is increasingly being used to achieve agricultural and environmental management objectives. Biochar increases pH, moisture-holding capacity, and nutrient retention in soil, providing a habitat for beneficial microorganisms, improving the cation exchange capacity, and reducing soil erosion [7]. Biochar has also been proven to trap carbon from the atmosphere and transport it to the soil [8]. The addition of biochar to concrete has also been proven to improve the mechanical properties of concrete [6]. The number of scientific studies related to biochar has risen exponentially in recent years, resulting in an expanding body of information regarding biochar and the pyrolysis process [9,10,11,12].

Our goal was to use the circular economy approach to utilize spent vetiver roots, initially used for oil extraction, to generate high-quality biochar. The objectives of this study were (i) to generate biochar from spent vetiver roots (after oil extraction) and unused vetiver shoots using different pyrolysis temperatures and holding times and (ii) to characterize the resulting biochars for their physicochemical characteristics to determine their suitability for use in environmental and agronomic applications.

## 2. Results and Discussion

### 2.1. Effect of Pyrolysis Temperature and Residence Time on the Physical Characteristics of Biochar

**Figure 1:**
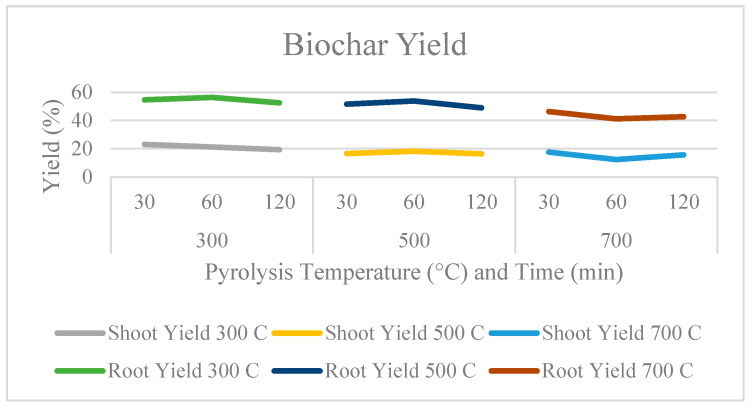

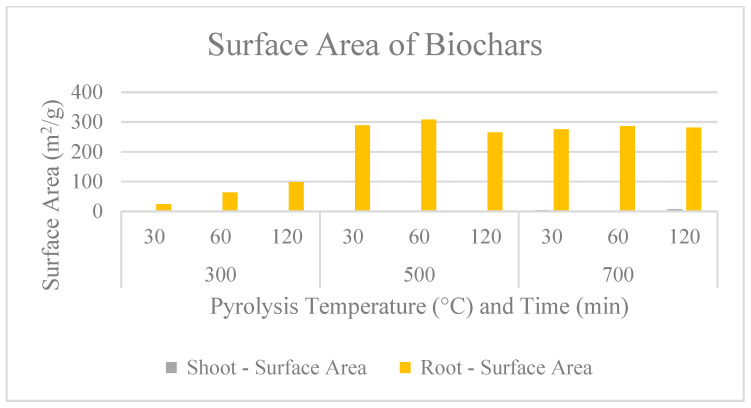
Yield and BET surface area of the shoot and root biochar from spent vetiver.

As shown in Figure 1, low-temperature pyrolysis yielded more biochar than high-temperature pyrolysis. The yields of vetiver shoot and root biochar dropped as the pyrolysis temperature rose. The physical characteristics of the resultant biochar were directly controlled by the chemical composition of the biomass source [13]. At temperatures exceeding 120 °C, organic matter starts to break down and releases chemically bound moisture. As a result, the concentrations of these ingredients may influence how reactive they are and how much the physical structure is altered during processing [14]. This might explain both the disparity in mass yield between the shoot and root yields and the decline in mass yield with rising pyrolysis temperature.

Biochemical analysis was performed on the shoots of the vetiver grass in an earlier study [5]. High levels of cellulose, hemicellulose, and lignin were detected [5]. The composition of vetiver grass is similar to many other C4 grasses. The nonmonotonic behavior at certain residence times results in increased yield due to more complete pyrolysis of volatile matter, but after a certain temperature, it might decrease due to further breakdown and gasification of the char material. Similarly, the BET surface area might initially increase as pores develop but then decrease if the prolonged exposure to heat leads to pore collapse or aggregation. Vetiver shoots generally have more cellulose and hemicellulose but less lignin compared to vetiver roots. Lignin-rich biomass tends to produce biochar with a higher surface area due to the formation of a more porous structure during pyrolysis. The lower lignin content in shoots leads to less pore development, resulting in a lower surface area. Vetiver root biochar produced at 500 °C for 60 min tends to have the optimal combination of complete carbonization and pore development while retaining structural integrity, leading to a higher surface area.

**Figure 2:**
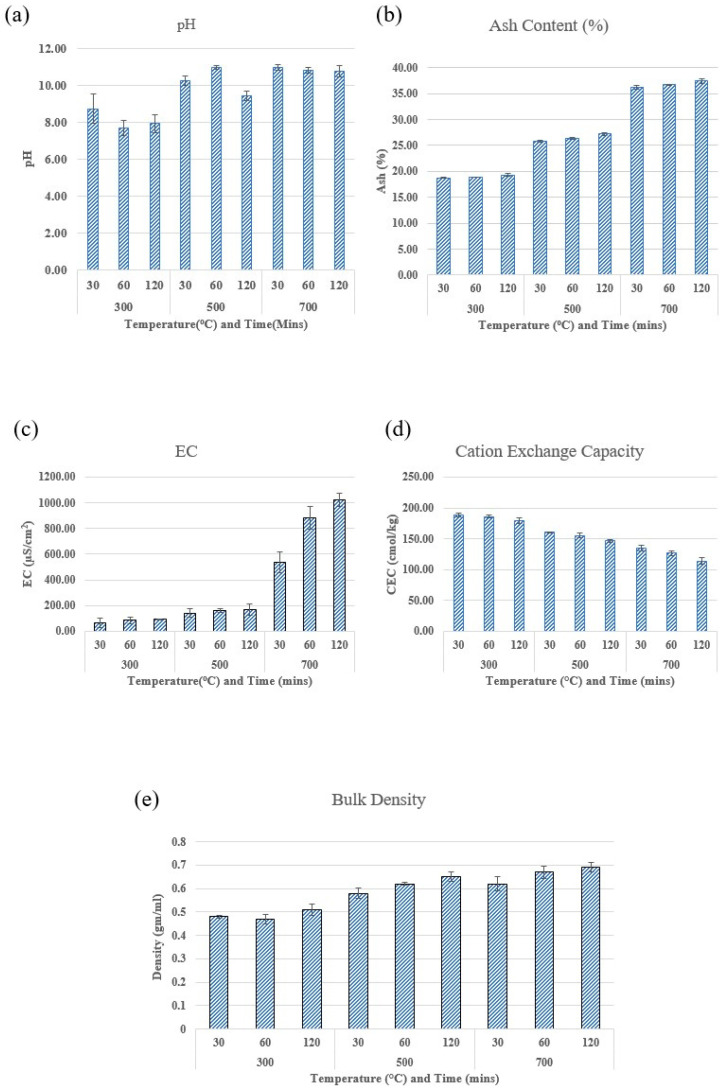
Physicochemical properties of vetiver root biochar. (**a**) pH, (**b**) ash content, (**c**) electric conductivity, (**d**) cation exchange capacity, and (**e**) bulk density.

The temperature at which the material was pyrolyzed had a significant influence on the pH of the biochar. The pH increased significantly (*p* < 0.05) with an increase in pyrolysis temperature from 300 to 700 °C, from less than 8 to 11, as seen in Figure 2a, likely due to the disintegration of the minerals from the organic framework and the rise in ash content with higher pyrolysis temperatures. Numerous factors, such as the proportion of inorganic components in the feedstock and the formation of basic surface oxides at high pyrolysis temperatures, can cause the pH to increase [15]. Varying pH values of the biochars might be explained by the variation in ash content. Ash concentration and pH measurements exhibited a positive correlation (r = +0.85, *p* < 0.05).

The majority of inorganic components do not vaporize at typical pyrolysis temperatures, which means that the mineral content in the feedstock largely influences the ash composition in biochar [16]. In all types of biochars, there was a notable increase in the ash content as the pyrolysis temperature increased. However, residence time did not have a significant impact on the biochar’s ash content at a given pyrolysis temperature. As illustrated in Figure 2b, the highest ash percentages were observed at elevated temperatures, exceeding 35% at 700 °C, while the lowest was found at 300 °C (below 20%). The increasing ash content in vetiver root biochar with rising temperature is primarily due to the thermal decomposition of organic components, leading to a corresponding increase in the inorganic fraction (ash), alongside possible transformations and stabilization of minerals that contribute to the ash content.

As pyrolysis temperatures and times increased, EC increased in a trend similar to that of pH and ash content, as seen in Figure 2c. One of the main determining elements, with a positive correlation (r = +0.9, *p* < 0.05), was the ash content. Electrical conductivity and the soil characteristics used to assess agricultural production are strongly connected. For instance, electrical conduction is made simpler when the overall soil porosity is higher. According to Rehrah et al. [17], adding biochar to the soil with a high enough EC value can help increase the fertility and quality of the soil.

Biochar produced at higher temperatures possesses an ash component with micronutrients that can aid in replenishing nutrient-deficient soils, thereby enhancing agricultural yields [17]. With an increase in pyrolysis temperature, the bulk density showed a tendency to rise (Figure 2e). At a certain temperature, residence time has little effect. Biochar generated at 700 °C and kept for 120 min has the maximum bulk density, according to previous reports. The bulk density may have an absolute impact on the soil’s composition. Biochar with low bulk density may promote soil porosity, minimize compaction [15], and improve root growth and air and water circulation. The growing carbon content is another factor contributing to the higher bulk density at high temperatures [18].

### 2.2. Effect of Pyrolysis Temperature and Residence Time on the Chemical Characteristics of Biochar

The CEC of biochar was significantly affected by pyrolysis temperatures and residence times. As can be observed in Figure 2d, the CEC decreased significantly (*p* < 0.05) as pyrolysis temperatures and residence times increased. According to Novak et al. [15], this is explained by an increase in the aromaticity of biochar and the loss of functional groups from its surface. According to Lu et al. [19], CEC decreases as the pyrolysis temperature increases because the bulk of active sites are stable during the thermal process. As a result, there are fewer oxygen-free Lewis base sites on the surface of biochar than there are acidic oxygen-containing surface groups.

Table 1 presents the elemental composition of biochar. The Sample ID can be read as “Sample Number-Pyrolysis Temperature-Pyrolysis Time”. With an increase in temperature, biochar’s carbon content increased while its hydrogen and oxygen content declined. The carbon content and biochar production were negatively correlated (r = −0.88, *p* < 0.05), which may indicate that carbonization accelerated as temperature increased throughout the pyrolysis process. High pyrolysis temperatures caused weak connections within the biochar structure to cleave and fracture, lowering the amount of hydrogen and oxygen present.

The maturity and aromaticity of the biochar is indicated by the O/C and H/C ratios. According to Kim et al. [20], the H/C ratio is negatively correlated with aromaticity, and a falling H/C ratio denotes a greater aromaticity level. On the other hand, according to Ahmad et al. [21], the O/C ratio represents the biochar’s moisture content and hydrophilicity. Long-chain organic molecules like lignin and cellulose are responsible for high carbon and oxygen content [22]. Figure S1 and Table S1 provide further details on the van Krevelen plot and the inorganic components of the biochar, respectively, in the Supplementary Information. The van Krevelen plot shows how its chemical structure becomes more aromatic and carbon-rich as it is subjected to higher temperatures, which is desirable for certain applications like carbon sequestration or soil amendment due to increased stability and decreased reactivity of the char. Table S1 lists the inorganic composition of the biochars generated at different temperatures. The decrease in heavy metal concentration with increasing temperature, despite a reduction in char yield, can be attributed to the volatilization of metals, chemical transformations, enhanced fragmentation of the biomass matrix, ash fusion and agglomeration processes, and the specific behavior of individual metals under high-temperature conditions.

Increasing pyrolysis temperatures were found to increase the liming value (LV) of biochar. The temperature fluctuations were not statistically significant (*p* > 0.05). The XRD spectra showed that calcite and other carbonate minerals were present, which was directly responsible for the growing trend. It was apparent that the LV could not be determined only by the pH of the char. As shown in Table 2, this vetiver root biochar performed well, in contrast to other C4 grasses [14]. To our knowledge, there are no studies on the use of vetiver root biochar as a liming agent on acidic soils. Additional studies are required to evaluate biochar’s capacity to neutralize acidic soil, particularly soils impacted by acid mine drainage.

### 2.3. Surface Characteristics

A significant (*p* < 0.05) increase in the total surface area of biochars occurred with rising temperatures, as seen in Figure 1. Vetiver roots are more suitable for pyrolysis than shoots, and the surface area of root biochars was significantly larger (*p* < 0.01) than that of the shoots. The biochar generated at 500 °C for 60 min had the highest surface area (308.15 m^2^/g). The composition of cellulose plays a key role in pyrolysis, as is clear from prior research, with cellulose microfibrils predominating the pyrolysis dimensional change process [23].

According to Joseph and Lehmann [14], the shrinkage that results from the aromatization process during the thermal breakdown of glycosidic chains is a result of the development of graphitic layers. In contrast to inorganic components (ash), lignocellulosic material components (lignin and cellulose) form biochar with the same level of microporosity [18]. All the physicochemical properties were solely evaluated for the biochar produced from the roots as it had a higher yield and surface area.

**Figure 3:**
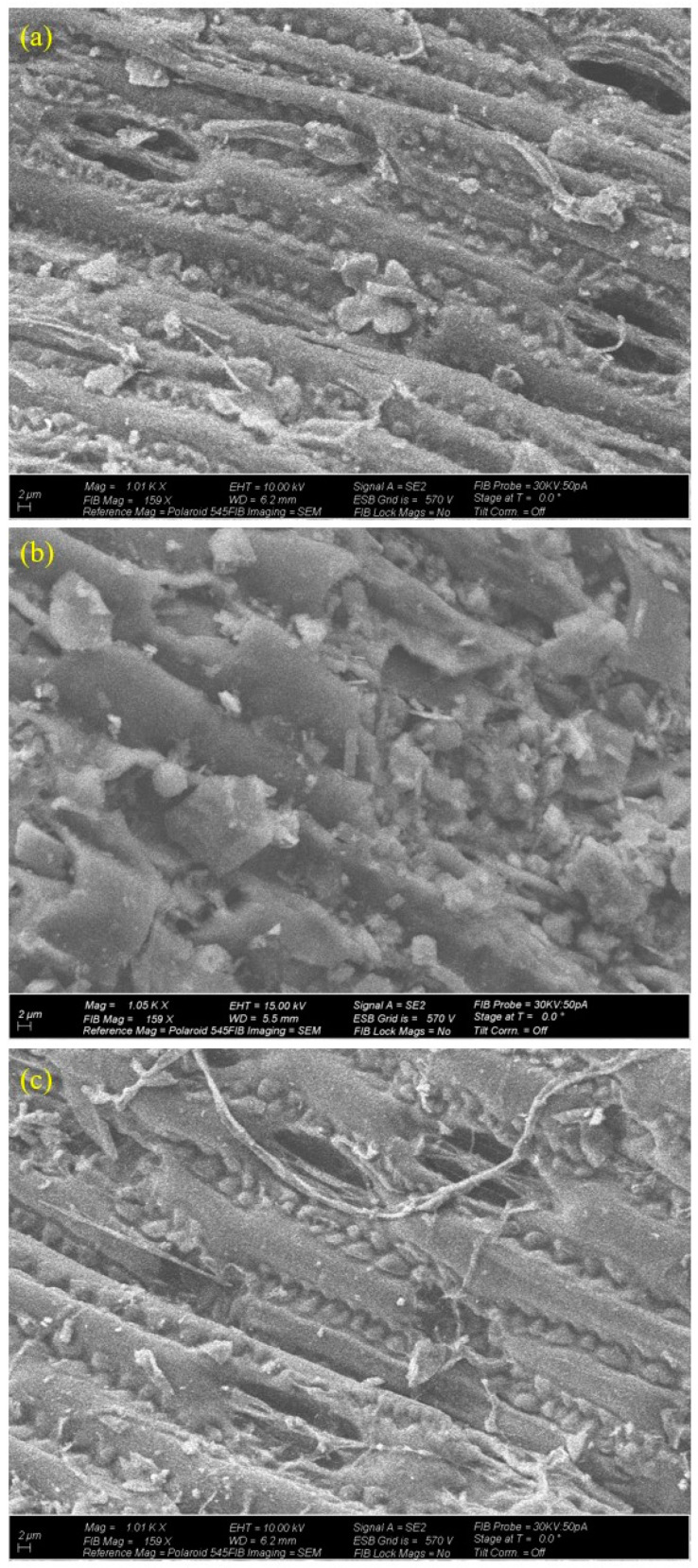
Spectroscopic analysis of vetiver biochar. Scanning electron microscope images for milled biochar particles: (**a**) SEM for 300 °C and 60 min, (**b**) SEM for 500 °C and 60 min, and (**c**) SEM for 700 °C and 60 min.

The topography and morphology of the generated biochar were examined using SEM images. The biochar had a nonuniform fibrous structure, as seen in Figure 3, with several pictures exhibiting tube-like pore spaces, approximately 5–10 µ in width, mimicking a honeycomb pattern. An enhanced SEM image with higher magnification is presented in the Supplementary Material. This kind of construction can enhance structurally and retain soil nutrients [22]. A comparison of the SEM images revealed that thermal treatment caused morphological changes as the porosity appeared to increase along with the pyrolysis temperature.

**Figure 4:**
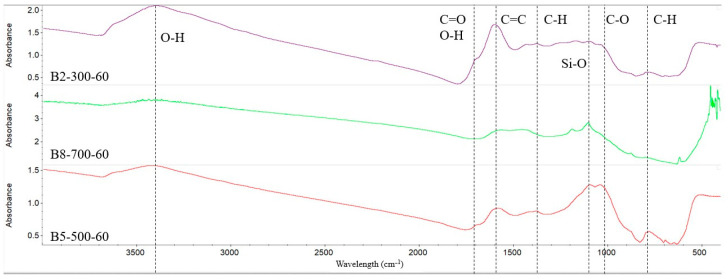
Fourier transform infrared (FTIR) spectra of the biochar at 300, 500, and 700 °C for 60 min each.

The FTIR spectra of all the biochar samples, as seen in Figure 4, revealed a broad band in the spectral region of 3300 to 3500 cm^−1^, which was attributed to -OH groups produced from H_2_O or phenolic compounds. Moisture loss at higher temperatures also explains why the sample’s peak at 700 °C was lower than those at 300 and 500 °C. All samples had aliphatic functional groups with assigned absorbance between 2920 and 2885 cm^−1^ (C-H stretching), although they were not discernible. According to Cantrell et al. [16], the strong band at 1030 cm^−1^ that comes from C-O stretching is associated with the oxygenated functional groups of cellulose, hemicellulose, and methoxyl groups of lignin.

**Figure 5:**
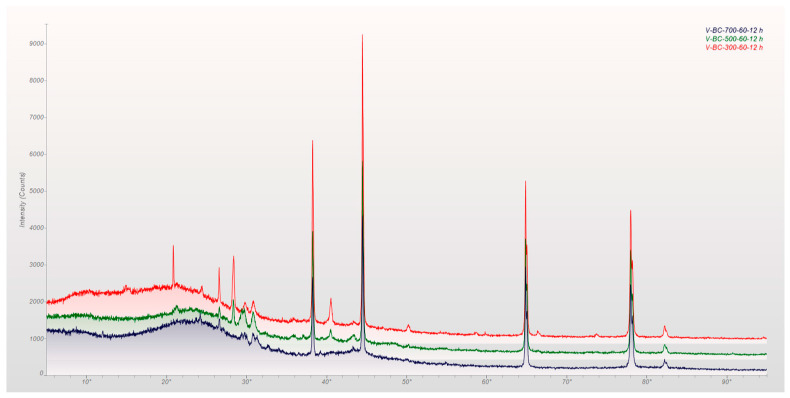
X-ray diffraction spectra of the biochar at 300, 500, and 700 °C for 60 min each.

The mineral components that were found as crystals in all of the root biochars were identified using X-ray diffraction. The spectra for the biochars at various temperatures sustained for 60 min are shown in the diffractogram in Figure 5. When biochar is produced at higher temperatures, as is the case with biochar produced at 500 and 700 °C, the volatile, amorphous phase peaks that are present when biochar is produced at lower temperatures disappear. The high Ca and Si content of the biochar, as shown by its elemental analysis, is compatible with the existence of calcite and quartz in the biochar. As a result of the interaction between monovalent cations and CO_2_ created during the thermal breakdown of hemicellulose and cellulose, merrillite, whewellite, and sodalite may have been formed more quickly [24].

Table 2 lists the main elements of vetiver biochar in comparison to other C4 grasses. All the mineral peak intensities decreased as pyrolysis temperatures increased, which is consistent with cellulose degradation [25]. At the same temperature, there were hardly any differences between the samples, but at various temperatures, the samples were distinguishable. Compared to powder diffraction and standards (PDF), these noticeable peaks in the biochars demonstrate the presence of inorganic components, including SiO_2_, KCl, and CaCO_3_. The presence of aluminum in the main peaks may be the result of scattering from the sample holder, which may be disregarded, while several unidentified peaks could not be located. Sylvite and calcite were also present in the C4 grasses studied by other researchers [25,26,27,28,29].

### 2.4. Environmental Implications of Vetiver Biochar

According to Kuzyakov et al. [30], the aromaticity and sequestration capacity of carbon in soils is indicated by the properties of biochar, such as its carbon content (% C), atomic ratios, low H/C ratios, and specific functional groups from its FTIR spectra. According to Xu et al. [31], 12% of global methane emissions are caused by agricultural soils, notably paddy rice soils. Agriculture has a considerable impact on greenhouse gas emissions in several subtropical nations, such as Brazil [24]. The findings of Cayuela et al. [32] show that the main factors affecting N_2_O emissions from biochar-amended soils include feedstock type, pyrolysis temperature and duration, soil properties, biochar dosage, and biochar and N source interactions.

Because of its strong affinity for sorption and resilience to microbial deterioration, biochar provides an essential binding phase for organic contaminants in the environment. Both carbonized and noncarbonized organic materials can be found in biochar. The ratio of carbonized to noncarbonized fractions as well as the surface and bulk qualities all affect the sorption capacity of biochar [33]. The surface area rose with rising temperature, which was also visible in the SEM pictures. New pore spaces make it possible for an ecosystem of microorganisms to grow, and their capacity to hold water increases.

Several studies have shown the heavy metal adsorption potential of vetiver root biochar at high concentrations in the aqueous media [34,35,36]. These studies have also demonstrated the effectiveness of vetiver root biochar to adsorb these metals in a coexisting matrix and its sustainable reuse as an admixture in green concrete [6]. The sorption of hydrophobic organic compounds by soil was enhanced by the addition of biochar, as demonstrated by Zhang et al. [37], although the extent of the improvement varied depending on the quantity of organic carbon already present in the soil, the method used to make the biochar, and the length of time it was in contact with the soil. Additionally, increased sorption may reduce soil contaminants’ mobility and hence prevent their leakage into groundwater. Organic pollutants (like POPs) exhibit substantial absorptivity for biochar due to their high affinity for natural or carbonaceous sorbents.

### 2.5. Agronomic Implications of Vetiver Biochar

The impact of biochar on soil density, porosity, particle size distribution, and soil structure has been shown in several studies. Due to its very porous composition and large surface area, biochar can help in the growth of beneficial soil microorganisms, such as mycorrhizae and bacteria. Biochars can also influence the binding of important nutritional cations and anions. Studies have shown that the application of vetiver root biochar as a conditioner for enhancing soil quality of copper-contaminated stamp sand from Michigan increased plant growth output, increased water retention, improved water quality, reduced nutrient leaching, reduced soil acidity, and met irrigation and fertilizer needs [38].

Alkaline compounds have a high ash content, which increases the soil’s capacity to buffer and neutralize acidity. This might partially replace the enormous volumes of limestone that are currently needed [24]. Additionally, biochar’s liming action, which elevates soil pH, has been shown to boost the availability of nutrients like N and P [39]. The high Ca and K contents in the biochars are evidence of their enormous agronomic utility, which may significantly replace conventional sources of K and Ca that are often provided by synthetic chemical fertilizers.

Despite the high overall concentrations of these chemical components in biochars, the availability of nutrients is a crucial factor to consider [40]. This is because an increase in pyrolysis temperature may cause certain nutrients in biochar to become less labile. The use of biochar in conjunction with N fertilizers has greatly enhanced the output of food crops [41]. The characteristics of vetiver biochar are comparable to previous reports that showed the use of biochar at greater concentrations resulted in substantial alterations in the soil’s quality, including increases in organic carbon, pH, and CEC as well as a reduction in tensile strength.

## 3. Materials and Methods

### 3.1. Vetiver Plants

Vetiver slips (8 to 10 inch long) were obtained from Mosquito Hawk Farms LLC, Anahuac, TX, USA. The slips were planted in pots containing garden soil and grown in a temperature-controlled greenhouse for 45–60 days. The watering was conducted using a sprinkler system, and a 14:10 h light/dark cycle was maintained. At the end of the growth period, the plants were harvested, and the roots were washed to remove the soil. Essential oils were extracted from the roots using hydrodistillation according to Martinez et al. [42]. The shoots were used to generate bioethanol using simultaneous saccharification and fermentation [5].

### 3.2. Vetiver Biochar Generation

To eliminate any remaining contamination, vetiver roots recovered from the process of extracting the oil were washed three times in succession with deionized (DI) water. The fine roots were dried at ambient room temperature for 2 days. In an SDT Q600 thermal analyzer, the thermal behavior and temperature profile of the roots were analyzed using thermogravimetric analysis (TGA), as shown in Figure S2. A sample of 10 mg of ground roots with a particle size less than 1 mm (passing Sieve No. 18) was analyzed with a temperature ranging from 25 to 1000 °C at a steady rate of 10 °C min^−1^ and a nitrogen flow rate of 100 mL min^−1^.

Based on the TGA data, a two-factor factorial design was utilized to produce biochar, with the first factor being pyrolysis temperature and the second being residence time. Three sets of pyrolysis temperatures (300, 500, and 700 °C) and residence times (30, 60, and 120 min) were used to design the experiment. Intermediate pyrolysis was chosen over low temperature and fast torrefaction as several studies have shown that the physicochemical characteristics of biochars are enhanced with increasing temperatures [21,43,44]. Biochar was generated in batches of 10 g of finely chopped biomass (shoots and roots), approximately 1 mm in size, and added to porcelain crucibles with lids (Cole-Palmer, Vernon Hills, IL, USA). The feedstocks were subjected to pyrolysis in a 51862 HR Lindberg furnace with a nitrogen gas retort (Watertown, WI, USA) at a flow rate of 0.1 mL min^−1^. The temperature was raised from the ambient temperature of 25 °C to the desired temperature at a rate of 10 °C/min. The biochars were ground in a ball mill according to Na Nagara et al. [45] with minor procedural modifications. Various physicochemical properties of the biochars were evaluated along with their elemental analysis and surface morphology.

### 3.3. Physical Properties

Biochar yield was determined as a percentage of the dry mass of the biochar over the dry mass of the roots before pyrolysis [46]:

Yield (%) = (Mass of biochar (g)/Mass of dry roots (g)) × 100(1)

Biochar was suspended in water heated to 90 °C at a 1% (*w*/*w*) ratio and stirred for 20 min, followed by measuring the pH of the solution using a pH meter. Then, 0.4 g of biochar was agitated in 40 mL of DI water at 27 ± 2 °C for 20 min [18] to measure the electrical conductivity (EC). Ash content in the biochar was determined using ceramic crucibles containing 2 g of oven-dried biochar, heated for at least six hours at 800 °C in a muffle furnace (Fisher Scientific, Hampton, NH, USA). After cooling, the residual solids were weighed [47]. The bulk density of the biochar was measured according to Ahmedna et al. [18] using the following formula:

Bulk density (g/mL) = (Mass of biochar (g))/(Volume of crucible (mL))(2)

### 3.4. Chemical Analysis

The cation exchange capacity (CEC) was analyzed using the sodium acetate method [48] with minor modifications. The variance between samples of biochar that were oven- and air-dried allowed the calculation of the water content correlation factor. A 50 mL centrifuge tube was filled with 15 mL of 1 M NaOAc (pH 8.2) and 0.2 g of the air-dried sample before being vortexed and centrifuged at 3000× *g* for 15 min. Instead of decanting, the supernatant was carefully removed using a pipette to prevent the loss of any particles. This step was repeated three times. The same process was used using 80% isopropanol, and the EC of the supernatant was measured before it was discarded. This process was carried out repeatedly until the conductivity fell below 1 µS/cm. The biochars were air-dried before being subjected to four separate reactions with 15 mL of 0.1 M NH_4_Cl. The total quantities of the supernatants were made to equal 100 mL after each collection. The solution was filtered using a 0.45 µm syringe filter before being subjected to an inductively coupled plasma optical emission spectrometry (ICP-OES) analysis to detect sodium (Na). The formula used for calculating the CEC was as follows:

CEC = (C × 0.435)/(W × F) (cmol/kg)(3)

where F = (oven-dried sample − air-dried sample), C = Na concentration (mg/L), and W = air-dried sample.

With a few minor adjustments, the standard EPA method (EPA SW 846 method 3050B; EPA, 1989) was used to determine the inorganic components of biochar [49]. First, 10 mL of 1:1 HNO_3_ was used to digest 0.5 g of each type of biochar at 95 °C for 1 h, followed by 5 mL of concentrated HNO_3_ for an additional hour. The sample was treated with 3 mL of 30% hydrogen peroxide. The sample was then refluxed with 2 mL of deionized water for 75 min. This solution was analyzed after filtering and dilution using ICP-OES (5100 Agilent Technologies, Santa Clara, CA, USA) for Al^2+^, Hg^2+^, As^2+^, Zn^2+^, Cd^2+^, Pb^2+^, Ni^2+^, Si^2+^, Cr^2+^, and Cu^2+^. The C, H, O, and N contents were analyzed with an elemental analyzer (Costech 4010, Valencia, CA, USA).

The liming value (LV) was calculated using the acid–base titration technique outlined by Joseph and Lehmann [14] with a few minor adjustments. First, 10 mL of 1 M HCl and 0.2 g of milled biochar were combined in a 50 mL centrifuge tube. The tubes were then kept standing overnight after two hours of stirring. The solution was titrated with a 0.5 M NaOH solution after the initial pH was recorded until the pH was reported at 7.0 along with a blank titration.

### 3.5. Surface Characterization

Based on the adsorption of nitrogen at 77 K, the surface area of biochar was calculated using Micromeritics ASAP 2020 (Micromeritics, Norcross, GA, USA). After being gassed overnight, a sample of biochar weighing between 0.2 and 0.25 g was added to the sample chamber. Results from Brunauer, Emmett, and Teller (BET) surface area and pore size distribution calculations were produced automatically by a Micromeritics tool. The following BET equation was used by the program to determine the specific surface area of biochars [50,51]:

V/V_m_ = C (P/P_0_)/[1 + (C − 1) (P/P_0_)] [1 − (P/P_0_)] (4)

The surface morphology of the biochars was examined using a scanning electron microscope–energy dispersive spectroscopy (SEM–EDS; Auriga Small Dual-Beam FIB-SEM, Carl Zeiss, Jena, Germany) and an 80 mm^2^ silicon drift detector EDS system (Oxford Instruments, Concord, MA, USA). Fourier transform infrared spectroscopy (FTIR Spectrometer, PerkinElmer Spectrum 100, Waltham, MA, USA), with spectra acquired between 400 and 4000 cm^−1^, was used to assess the surface chemical characteristics of the biochars. Sample pellets with a 1:100 biochar to KBr ratio were prepared for transmission tests. For each sample, an average of 100 images with a spectral resolution of 4 cm^−1^ were processed. The chromatograms were analyzed using OMNIC v9.2 software and FTIR from Thermo Fisher Scientific Inc., Waltham, MA, USA. An X-ray powder diffractometer (Scintag XDS 2000, SPW Industrial, Laguna Hills, CA) with a Cu Kα radiation source at the anode (45 kV/35 mA, λ = 1.5406) was used to examine the X-ray diffraction (XRD) of biochar with a scan rate of 12 s per step. The samples were examined in an angular 2θ range of 5 to 95° with the aid of MDI Jade^®^ Version 8.5, which features an ICDD PDF2 inorganic database.

### 3.6. Chemical and Statistical Analysis

Certified reference solutions were verified in addition to the biochar solution sample. Both internal (Y) and external standards were examined in every ICP-OES analysis. Standard solutions with R^2^ > 0.995 were established before each study to calibrate the system. Three replicated measurements were made for every sample. ICP Expert Software v7.1.0.6821 (Agilent Technologies, Santa Clara, CA, USA) was used to analyze the ICP-OES data. All the containers utilized in the tests were washed with laboratory-grade detergent, steeped in a 10% nitric acid solution for the duration of the night, then flooded with deionized (DI) water before receiving a final rinse with DI water. Significant differences (*p* < 0.05) and the Tukey–Kramer honestly significant difference test were evaluated using the statistical tool JMP 15.2 (SAS Institute Inc., Cary, NC, USA). The Origin 2022b statistical program (OriginPro, Version 2022b, OriginLab Corporation, Northampton, MA, USA) was used to create data visualizations.

## 4. Conclusions

This study demonstrates that a circular economy model is an efficient solution to prevent the waste stream from going to landfills. Vetiver roots used for oil extraction can be optimally converted into a “green” biochar. Compared to the shoots, vetiver roots produced biochar with a significantly larger surface area. The yield of biochar decreased as the pyrolysis temperature rose, and the biochar’s physicochemical and structural characteristics were considerably altered. Analysis showed that vetiver root biochar generated at 500 °C and held for 60 min had the highest surface area of 308.15 m^2^/g and a yield of 53.76%. As the temperature rose, the pH, EC, ash content, bulk density, and carbon all increased, but the CEC had a downward trend. The biochar’s liming value increased along with the temperature. Vetiver root biochar presents a multitude of agronomic and environmental applications because of its aromaticity with prospects of enhancing the quality of the biochar through activation, source modification, formulations, etc. Further research can also investigate the sequestration potential for various greenhouse gas emissions as a promising carbon capture and storage technology.

## Figures and Tables

**Table 1:** Elemental composition of spent vetiver root biochar.

Sample ID	Temperature (°C)	Retention Time (min)	%C	%H	%N	%O	H/C	O/C	%P	%K	%Ca	%Si
B1-300-30	300	30	61.11	3.945	2.102	21.7	0.065	0.355	0.006	0.012	0.019	0.025
B2-300-60	300	60	63.75	4.056	2.092	24	0.064	0.377	0.008	0.013	0.021	0.029
B3-300-120	300	120	61.99	3.9	2.033	23	0.063	0.371	0.010	0.016	0.025	0.03
B4-500-30	500	30	62.32	2.501	1.256	15.8	0.040	0.254	0.024	0.029	0.026	0.032
B5-500-60	500	60	68.58	2.782	1.752	20.7	0.041	0.302	0.029	0.032	0.027	0.034
B6-500-120	500	120	66.24	3.239	2.092	20.7	0.049	0.313	0.036	0.038	0.031	0.038
B7-700-30	700	30	73.51	1.135	0.432	15.6	0.015	0.212	0.051	0.059	0.035	0.041
B8-700-60	700	60	71.20	0.969	0.198	15	0.014	0.211	0.053	0.068	0.038	0.041
B9-700-120	700	120	84.62	1.596	0.266	14	0.019	0.165	0.057	0.072	0.043	0.05

**Table 2:** Mineral composition of vetiver root biochar compared to other C4 grasses.

Feedstock (Temp °C)	Crystalline Phases	Liming Value (% CaCO_3_)	
		Mean	Std. Error
Vetiver root (300)	Quartz, calcite, merrillite	2.54	0.31
Vetiver root (500)	Quartz, whewellite, sylvite	3.7	0.67
Vetiver root (700)	Quartz, arcanite, sylvite	5.76	0.08
Wheat straw (550)	Quartz, calcite, Mg calcite	5.7	0.1
Wheat straw (700)	Quartz, calcite, Mg calcite	6.5	0.1
Switchgrass (400)	Quartz	1.9	0.2
Switchgrass (550)	Quartz	3	0.2
Rice husk (550)	Quartz, calcite	1.5	0
Rice husk (700)	Quartz, calcite, sylvite	1.9	0.1
Miscanthus straw (550)	Quartz, calcite	3.8	0.1
Miscanthus straw (700)	Arcanite, calcite	5.6	0.2

## Data Availability

Data are contained within the article and Supplementary Materials.

## Supplementary Materials

The following supporting information can be downloaded at: https://www.mdpi.com/article/10.3390/molecules29010063/s1. Figure S1: The van Krevelen plot of biochar produced at 300, 500, and 700 °C. Table S1: Elemental composition of vetiver root biochar. Figure S2: Thermogravimetric (TG) and derivative thermogravimetric (DTG) analysis for vetiver roots. Figure S3. Spectroscopic analysis of vetiver biochar. Scanning electron microscope images for milled biochar particles: (a) SEM for 300 °C and 60 min, (b) SEM for 500 °C and 60 min, and (c) SEM for 700 °C and 60 min.
