# Supplementary material for: Valorization of Spent Vetiver Roots for Biochar Generation
Source: Molecules. 2023 Dec 21;29(1):63. doi: 10.3390/molecules29010063 (PMC10779468; doi:10.3390/molecules29010063)
Supplement: Supplementary file 1 [file molecules-29-00063-s001.zip › molecules-2699719-supplementary.pdf]

# Valorization of Spent Vetiver Roots for Biochar Generation

**Sameer Neve [1], Dibyendu Sarkar [1,*], Manas Warke [2], Teresa Bandosz [3] and Rupali Datta [2]**

[1] Department of Civil, Environmental, and Ocean Engineering, Stevens Institute of Technology, Hoboken, NJ 07030, USA; sneve@stevens.edu
[2] Department of Biological Sciences, Michigan Technological University, Houghton, MI 49931, USA; mwarke@mtu.edu (M.W.); rupdatta@mtu.edu (R.D.)
[3] Department of Chemistry, City College of New York, New York, NY 10031, USA; tbandosz@ccny.cuny.edu
* Correspondence: dsarkar@stevens.edu; Tel.: +1-201-216-8028

## Supplementary Information

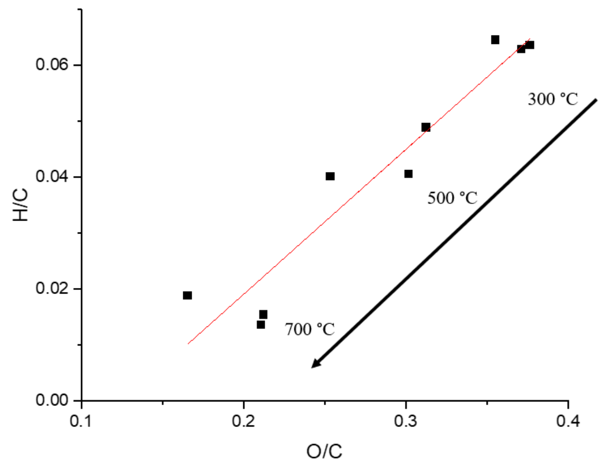


**Figure S1**: The Van Krevelen plot of biochar produced at 300, 500, and 700°C.
Van Krevelen plot was generated using the atomic ratios of H/C and O/C of biochar produced at varied pyrolysis temperatures. At higher biochar production temperatures, the atomic ratios H/C and O/C were lower.

**Table S1:** Elemental Composition of Vetiver Root Biochar

| Sample ID | Hg | As | Zn | Cd | Pb | Ni | Cr | Cu |
|---|---|---|---|---|---|---|---|---|
| | **μg/kg** | | | | | | | |
| **B1-300-30** | 0.232 | 0.053 | 14.62 | 0.155 | 0.183 | 5.678 | 1.855 | 10.62 |
| **B2-300-60** | 0.206 | 0.040 | 11.98 | 0.147 | 0.177 | 4.446 | 1.826 | 7.592 |
| **B3-300-120** | 0.204 | 0.032 | 9.449 | 0.139 | 0.136 | 1.795 | 1.644 | 7.365 |
| **B4-500-30** | 0.193 | 0.018 | 9.312 | 0.121 | 0.127 | 1.743 | 0.920 | 7.003 |
| **B5-500-60** | 0.191 | 0.016 | 7.706 | 0.086 | 0.126 | 1.622 | 0.862 | 5.127 |
| **B6-500-120** | 0.190 | 0.015 | 6.947 | 0.075 | 0.106 | 0.876 | 0.725 | 4.667 |
| **B7-700-30** | 0.186 | 0.010 | 4.664 | 0.072 | 0.100 | 0.824 | 0.722 | 4.622 |
| **B8-700-60** | 0.186 | 0.009 | 4.336 | 0.068 | 0.097 | 0.341 | 0.673 | 4.522 |
| **B9-700-120** | 0.165 | 0.008 | 3.795 | 0.064 | 0.076 | 0.294 | 0.642 | 4.254 |

Biochars were prepared at three temperatures – 300, 500, and 700$^{o}$C and three retention times – 30, 60, and 90 min. identified as B1 through B9.

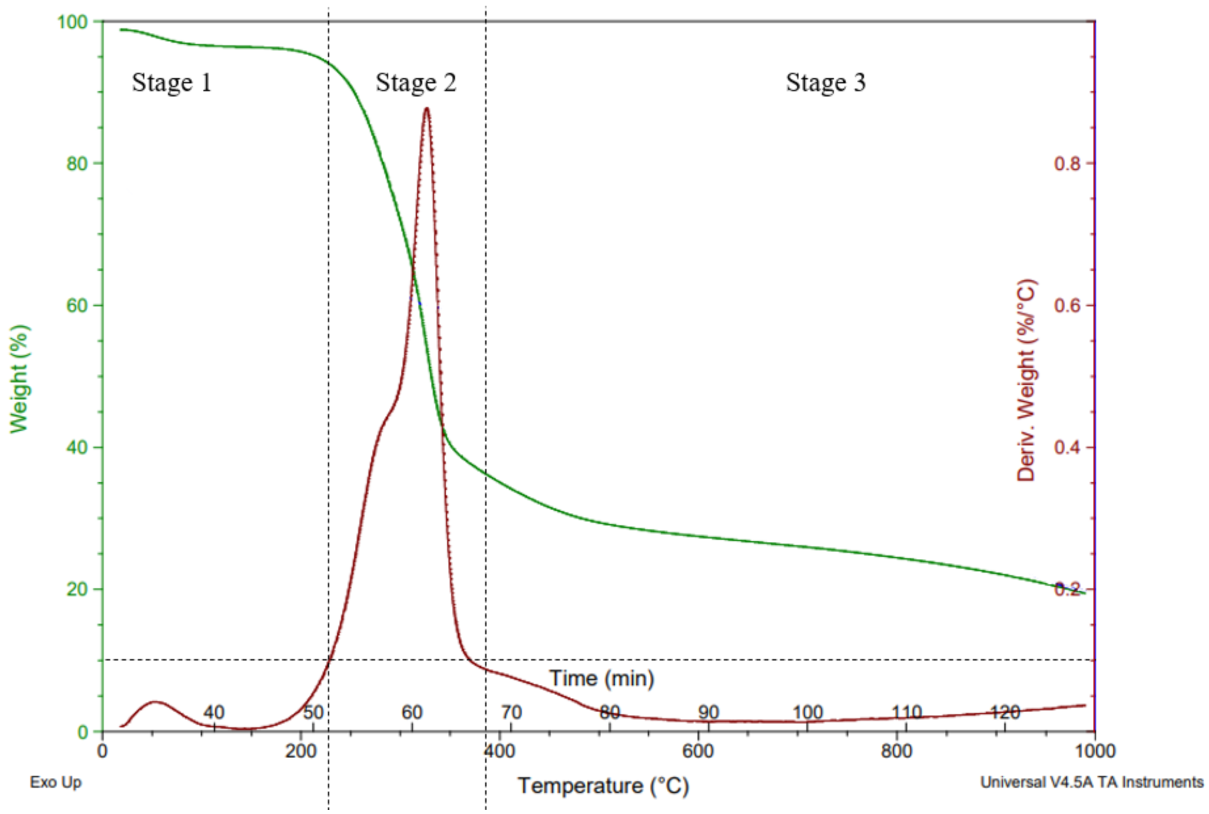


**Figure S2:** Thermogravimetric (TG) and derivative thermogravimetric (DTG) analysis for Vetiver Roots. Weight loss and derivative weight loss of the roots at heating rates of 10°C $min^{-1}$ through a temperature range of 25 to 1000°C. The whole mass loss curves for the roots can be separated into three stages, as shown in the diagram.

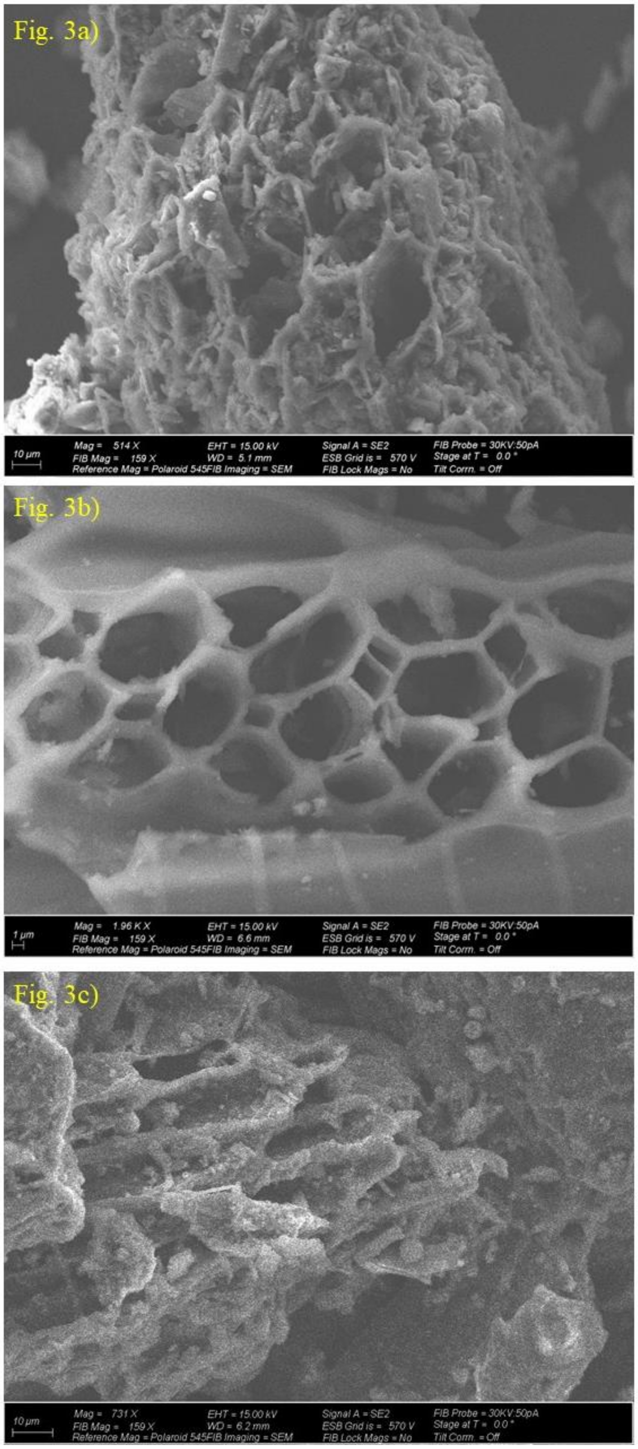


Figure S3. Spectroscopic analysis of vetiver biochar: Scanning Electron Microscope images for milled biochar particles: a) SEM for 300 °C - 60 min; b) SEM for 500 °C - 60 min; c) SEM for 700 °C - 60 min.
